# Editorial

**Published:** 1899-12

**Authors:** 


### TEXAS MEDICAL JOURNAL.

AUSTIN, TEXAS.

A MONTHLY JOURNAL OF MEDICINE AND SURGERY.

F. E. DANIEL, M. D., Editor.
S. E. HUDSON, M. D., Managing Editor.

Published Monthly at Austin, Texas, by Drs. Daniel and Hudson. Subscription price $1.00 a year in advance.

Eastern Representative: John Guy Monihan, St. Paul Building, 220 Broadway, New York City.

Official organ of the West Texas Medical Association, the Houston District Medical Association, the Austin District Medical Society, the Brazos Valley Medical Association, the Galveston County Medical Society, and several others.

### EDITORIAL.

**AN IMPORTANT DECISION.**

The Medical Practice Law in Texas.—There is a general misunderstanding among the medical men of the State and the more recent graduates, especially, as to the requirements of the existing law relative to the practice of medicine. The Journal receives numerous letters asking for information on the subject, and as it is impossible to answer all of these inquiries satisfactorily we referred the matter to our immediate representative in the lower house of the Texas Legislature, Hon. J. W. Maxwell, attorney-at-law, and give below his reply.

Office, Texas Medical Journal,
Austin, Texas, Nov. 6, 1899.

*J. W. Maxwell, Esq., Attorney-at-Law, Austin, Texas.*

Dear Sir: Will you kindly answer the following questions relative to the law in this State governing the practice of medicine?:

1. What constitutes a "legal practitioner of medicine" in this State?

2. Does the law require a graduate of "an accredited medical college duly chartered by the State in which it is located," and who holds a diploma from such college, and which diploma has been duly registered in the office of the court of the district in which such physician resides, to obtain a certificate from any board, as a condition to collecting his fees by law?

3. Is there any decision of any court in this State bearing on this point? The "Kennedy case" (see TEXAS MEDICAL JOURNAL, January, 1896, pages 382-390), seems to have been taken as a precedent, and physicians are writing the JOURNAL that they are being coerced before boards of examinations. Kennedy had not registered his diploma. Your reply will be published in the JOURNAL.

Yours truly,
EDITOR TEXAS MEDICAL JOURNAL.

MR. MAXWELL'S REPLY.

AUSTIN, TEXAS, December 1, 1899.

*Editor Texas Medical Journal:*

DEAR SIR: There seems to be an inconsistency between the provisions of the Penal Code and those of the civil statutes. The former does not prohibit one from practicing his profession who has received a diploma from "some accredited medical college, chartered by the State or its authority in which the same is situated," and who has had such diploma recorded as is required in Article 3787 of the Revised Statutes of 1895. Nor is there such a prohibition in the civil statutes. Yet the provisions of the civil statutes would seem to indicate that a certificate from a board of examiners would be necessary to make one a legal practitioner.

The leading case reported in this State, bearing on this subject, seems to be the case of Kennedy vs. Schoultz, reported in the Southwestern Reporter, Vol. 25, page 667. In this case, it was held that Kennedy held a diploma from a reputable medical college recognized as such by the American Medical Association, but as he did not have his diploma recorded in the office of the district clerk, he could not recover fees for professional services rendered. Justice Fly, in rendering his opinion, says: "While a physician must violate the civil statutes to practice medicine, even when he has a diploma, without first having obtained a certificate from a board of medical examiners, yet under the criminal statutes it would seem that a diploma from a chartered medical college would exempt from punishment under Penal Code, Article 396 (Statutes of 1879), but under Article 398 it is a violation of law to fail to record either." Kennedy contended that there was no board of examiners in the county in which he resided, and for that reason he could not obtain a certificate, but the court in the same case held, "To practice medicine without having obtained a certificate, and without having it duly recorded, is in violation of the civil statutes, and this failure cannot be excused on the ground that no board of medical examiners had been appointed. A violation of the law cannot be excused on the grounds that some one else has violated it, and if there was no board of examiners in Bexar county, as required by law, the appellant should have applied to some other board of examiners."

This opinion was reaffirmed in the case of Wilson vs. Vick, from Lee county, in an opinion rendered by Chief Justice Fisher, in the Court of Civil Appeals, and reported in the Fifty-first Southwestern Reporter, page 45. Wilson was a regular practicing physician, entitled to practice his profession by virtue of a regular diploma granted to him by the Vanderbilt University of Nashville, Tenn., that institution being a medical college recognized by the American Medical Association; and before he engaged in the practice of medicine, he caused said diploma to be recorded in the office of the district clerk of Lee county, Texas. In this case the court says: "There was no error in the judgment in this case, because the facts do not show that the appellant has received a certificate authorizing him to practice medicine, and that the same has been recorded as is required by the statutes."

The case of Wilson vs. Vick was carried to the Supreme Court on a writ of error, and there, by a recent opinion by Chief Justice Gaines (which opinion was rendered November 9, 1899, but has not yet been reported), was overruled and the cause remanded. This last opinion decides that Wilson was entitled to recover fees for professional services. Chief Justice Gaines, in commenting on the opinion of Justice Fly, in the case of Kennedy vs. Schoultz, says: "We are inclined to think that the decision properly construed the law as it then existed. The case arose before the adoption of the Revised Statutes of 1895. The Revised Statutes of 1879 contained this important provision: 'Article 3638. No person, except those named in the preceding article, shall be permitted to practice medicine in any of its branches or departments without first having obtained and recorded a certificate of qualification from some authorized board of medical examiners, as heretofore provided; and any person so offending shall be punished as provided in the Penal Code.' Physicians having diplomas are not among those excepted by the preceding article. This clearly makes practicing without a certificate unlawful. This article is wholly omitted from the Revised Statutes now in force. We are to presume that the omission was for a purpose and not inadvertent. That purpose would seem to be to bring the provisions of the civil statutes in consonance with the criminal statutes upon the same subject matter and to make it not unlawful for a physician having a duly recorded diploma from a proper college to practice his profession."

It seems, therefore, a settled question that one who has received a diploma from some accredited college, chartered by the State in which it is located, and who has had such diploma recorded, is entitled to collect his fees, and have all the rights and privileges under our laws, as well as one who has successfully stood the required examination before a board of examiners and received a certificate, and had the same recorded.

After briefly reviewing the authorities on this question, I think it is now well settled law that a legal practitioner of medicine, is either one who holds a diploma from an accredited medical college, chartered by the State or its authority in which the same is located, and who has had such diploma recorded, as is required by Article 3787, Revised Statutes, 1895, or one who has received a certificate from a board of medical examiners, as is required in Title LXXXII, of the Revised Statutes of 1895, and has had such certificate legally recorded, and in either case fees can be collected for professional services rendered.

Yours truly,

J. W. Maxwell.

---

**ONLY A DIPLOMA REQUIRED.**

"*'Tis true, 'tis pity, and pity 'tis 'tis true*," or words to that effect. It will be seen by reference to Mr. Maxwell's letter in the editorial columns that, as the Journal has insisted, a diploma, duly registered, is accepted in Texas as the equivalent of a license to practice medicine. The State Medical Association and the Journal have tried for years to get the law so changed as to knock out the diploma and require an examination as a condition to the exercise of the grave and dangerous privilege of practicing medicine, but without success or encouragement to try again. In fact, the Association has been laughed at, and its representatives who have appeared before the Legislature from time to time have been snubbed. Political influences control everything in this State, and the doctors have not had the pull, or have not pulled the right string. It is very remarkable that a self-constituted "faculty" is allowed to *license* men and women to practice medicine; to exercise a prerogative which belongs alone to the government, and especially when that "faculty" represent a medical school in another State—any old State. We all know that a diploma is not always evidence of qualification, even from the highest grade schools. And we all know, too, that the majority of medical colleges are individual enterprises, run to make money, and that almost anybody, anywhere, can get a charter for a few dollars, to establish a medical college. Nevertheless, all diplomas go in Texas; are taken as the equivalent of, and accepted as, a license. The law does stipulate that the diploma shall be from a medical college chartered in the State in which it is situated; but as the law does not make it anybody's business to see after diplomas and verify these things, even if they amounted to anything, but leaves it to the clerk of the court where the diploma is presented for record, it will readily be seen that the whole thing is a farce. What does the clerk of the court know about colleges, or care? So that he gets his dollar, there is no discrimination made nor attempted nor possible. Hence "everything goes" in Texas, more's the pity.

Nor is the system (?) of licensing by certificate from medical boards any less a farce. The district boards so paraded in this law are appointed, if appointed at all, for personal or political reasons, and with neither knowledge of the fitness of the appointees nor regard therefor, and hence in some districts there are boards not only incompetent but corrupt, and an examination is but a matter of dollars and cents with them. In certain districts, the JOURNAL is reliably informed, the boards make a discount to "lots of five" or more presenting themselves at one time, and this discount induced a lot of first-course students at one of the Texas colleges to bolt the school and get "certificates." Why grind out three or four years in hard study to get a diploma, when one can get a "certificate" from the board in an adjacent county? There are also boards who issue certificates "while you wait." In north Texas there are (or were) members of such boards, so we are informed, who carry in their pockets certificates signed and sealed, and all ready for delivery to any chance "applicant" except filling in his name. This is a lamentable state of things, but what are you going to do about it? We are pleased to say, however, that there are boards composed of capable and conscientious gentlemen, who earnestly strive to protect the people and the profession from incompetents. But their efforts are powerless so long as a rejected applicant can go to another district and practically buy a "certificate" for fifteen dollars. The clerk will record it, of course, and so, that fellow is "heeled" for all time. Is it a wonder that quackery is triumphant in Texas?

The situation demands that a State Board of Medical Examiners—competent and honest men—should be created by law, and nothing should be recognized but a license from this board.

A PUBLIC HEALTH DEPARTMENT. Early in January there will be held in Washington a meeting of representatives of every State Medical Association in America, the object of which is to make a personal appeal to Congress for legislation in the interest of the public health. To this end the delegation, two from each State Medical Association, will appear before a committee of the United States Senate and represent the need of action by the general government looking to the institution of sanitary measures for the protection of the people from such outrage, for instance, as we learn is about to be committed by Chicago in the matter of converting the Mississippi river into a great sewer, as pointed out in these pages last month; and for the protection of streams in general, and other water supply from pollution; protection of the people from the dangers of food adulteration, drug adulteration, the indiscriminate sale of poisons, the evils of unsanitary plumbing, etc., and especially to urge the institution by Congress of a uniform system of collecting and preserving the vital and mortuary statistics of the United States. In short, to induce Congress, if possible, to create a Department of Public Health, in lieu of the Marine Hospital "Bureau," an inefficient sort of side show to the Treasury Department. Another matter, of equal importance, should engage the attention of the delegation and Congress: the institution of a uniform system of license to practice medicine in lieu of the farce of recognizing a "diploma."

A PULL ALL TOGETHER.

Action by the general government is imperative, seeing that in many States the medical profession have never succeeded in impressing upon the Governor and State Legislature the importance and necessity of moving in the matter. Texas, especially, seems to be burdened with a political incubus, and her lawmakers and executive are so much under the domination of predecessors and traditions, that they cannot be induced to make any improvement. I am reminded in this matter of the old farmer who carried his corn to mill in one end of the sack and balanced it with stones,—as his father and grandfather had done,—and when his son suggested to throw away the stones and divide the corn, his father told him he was a fool. An outgoing Governor in his farewell message to the Texas Legislature, went out of his way to advise that no change be made in the Texas "public health laws,"* and the advice was religiously adhered to. His successor, in his message, accordingly made no

*We have no public health laws in Texas;—have nothing but a very primitive quarantine law.—ED.

recommendation as to medical or sanitary legislation, notwithstanding he was, as we are assured by the parties, fully committed to medical gentlemen in the State to do so, and to that end he had expressed a desire to have a consensus of medical opinion as to what changes were needed. The matter was laid before him by a select committee from the State Medical Association in person; laid before him also in print, and later, when it was seen that he had neglected the matter and the Legislature was close to adjournment, in the form of a memorial from the State Medical Association then in session, to none of which he paid the least attention.

Hence it is evident that Texas at least will do nothing for the better protection of her people, and an appeal to Congress is in order. It would seem that no argument would be needed to convince any one who pretends to be "an enlightened statesman" (and there are some who do so pretend), that the public health is the paramount consideration in all government. Upon its protection depend all others,--the wealth, strength and prosperity of the nation.

The meeting of these medical delegates in Washington will be one of great interest, and the results will be looked for anxiously by every one who feels an interest in the great subject. Only the very best men should be sent from each society, and no doubt the selections will be made with discriminating judgment. To date we have not been advised of the appointment of delegates by our own State Medical Association, but feel assured that our president fully realizes the importance of a judicious selection. That the United States of America has no system of vital statistics and no measures of protection of the people from indigenous diseases, easily preventable, is a reproach which it is hoped Congress will speedily wipe out.

---

**THE GOOD DIE YOUNG.**

"I KNEW—I knew—it could not last; 'twas bright, 'twas,"—well, it's gone up. We regret to announce the demise of our sprightly and plucky little Houston contemporary, *The Southwestern Medical and Surgical Record.* After life's fitful fever, let us hope "it sleeps well." Alas, that all things fair and bright should fade. *The Southwestern Medical and Surgical Record* did some good work, and was always looked for among our exchanges, and welcome. Its failure was in consequence of the lack of substantial appreciation at the hands of the Texas medical profession, to whose interests it was entirely devoted. It does not in any sense illustrate the survival of the fit,—for it deserved better support than it received. We shall miss it. Hence these tears.

**HIT IT AT LAST.**

AT THE request of the Surgeon-General of the Marine Hospital Service the President has detailed medical officers from that service to be attached to every consulate in the East. That is getting at the root of the matter, and if the duties for which these gentlemen are sent out are properly performed, no ship infected, or having on board any infected person or thing, will be permitted to sail for the United States. This sensible action has been too long delayed, we fear, and it may be a case of putting up the gap after the cow has gone out, seeing that already a ship has arrived at New York with two cases of bubonic plague on board. Better late, however, than never. Such service, if efficient, will be worth a thousand quarantines.

---

A TEXAS DOCTOR WHO KNOWS HOW TO ENTERTAIN, and does it, is Dr. J. W. Hunter of Waco, Texas, the traveling representative of the well known house of Keasbey & Mattison Co. Nearly all Texas, and most Southern doctors know Dr. Hunter, and they know him to be one of the most pleasant and affable gentlemen as well as the hardest working and most enthusiastic knight of the road. But to know him well and truly you must fall in with him when you are among strangers. During and following the recent meeting of the Mississippi Valley Medical Association in Chicago, it was the good fortune of the writer and his better half to meet up with and be entertained by Dr. Hunter. Nothing that he could do to increase our comfort was left undone, and on the day of the laying of the corner stone of the Chicago federal building we, together with some other ladies and gentlemen, were invited to his room—an elegant one on the sixth floor of the Great Northern Hotel, where we had a splendid view of the corner stone laying and the reviewing stand occupied by President McKinley. From this room we witnessed the great military and civic parade during the afternoon, and the parade of all nations at night. Dr. Hunter, so thoughtful of the comfort of his guests, had provided the most delicate and dainty refreshments, including cakes, lemonades, Gunther's finest candies, and (for the gentlemen) "Hunter's Best," etc. When the military and civic parade had passed, the entire party was invited to the Great Northern Café, where dinner, a most elegant one, was served. After we had spent almost the entire day and until ten-thirty at night as the doctor's guests, and had been royally entertained, not only in the matter of a hearty welcome and good cheer, but with many of the doctor's funny jokes and laughable experiences, he was heartily and unanimously voted the best entertainer on earth. When we had bidden the old doctor good night and passed out of the hotel, one of the ladies of our party remarked, that Dr. Hunter had done more to entertain his guests and had been more careful of their every comfort than any one she had ever known; and do you know, she said, "that I believe not one of us has enjoyed the day more than he has; he has been happy in the happiness of others." And she was right. S. E. H.

---

Yellow Fever in 1899 in the South. From the Marine Hospital Reports we compile the following: In Florida, from August 31 to November 15, 1433 cases; 76 deaths. In Louisiana (New Orleans), from August 27 to November 10, 113 cases; 20 deaths. In Mississippi, from September 1 to November 1, 88 cases; 11 deaths. Total cases, 1634; total deaths, 107. Percentage of deaths on total, 6.5. Percentage of deaths in Florida, 5.3; percentage of deaths in New Orleans, 17.7; percentage of deaths in Mississippi, 12.5.

Wide range from 5 per cent. to 17.7. Better doctors in Florida?

---

The Substitutor. It is very gratifying to note (see article elsewhere from *Chicago Times-Herald*), that one substituting druggist who puts in one's prescriptions "something just as good" has come to grief through the activity of Fairchild Brothers & Foster. This fellow was substituting his own manufacture for "Fairchild's Essence of Pepsine," and it cost him $500. It is a pity that such conduct is not a penal offense.

---

Physician's Pocket Manual. A compact, useful and handsomely printed book containing valuable and useful information about pharmaceutical and biological remedies. A complete property and Dose List; metric equivalents, botanical synonyms, formulas, etc. Parke, Davis & Co., Detroit, Mich., will send a copy free to any of our readers who mention this notice.

---

Dr. Daniel's book will soon be ready for delivery. Send in your orders now. Edition very limited. Price, postpaid, $1.10.

---

The Southern Texas Medical Association will meet in Houston December 13 and 14 (inst.).

